# A mixed‐method systematic review and meta‐analysis of mental health professionals' attitudes toward smoking and smoking cessation among people with mental illnesses

**DOI:** 10.1111/add.13387

**Published:** 2016-05-03

**Authors:** Kate Sheals, Ildiko Tombor, Ann McNeill, Lion Shahab

**Affiliations:** ^1^ Department of Epidemiology and Public HealthUniversity College LondonLondonUK; ^2^ Institute of Psychiatry, Psychology and Neuroscience UK Centre for Tobacco and Alcohol Studies, London, UKKing's College LondonLondonUK

**Keywords:** Attitudes, health care professionals, mental health, meta‐analysis, psychiatric patients, systematic review, tobacco treatment

## Abstract

**Background and aims:**

People with mental illnesses and substance abuse disorders are important targets for smoking cessation interventions. Mental health professionals (MHPs) are ideally placed to deliver interventions, but their attitudes may prevent this. This systematic review therefore aimed to identify and estimate quantitatively MHPs attitudes towards smoking and main barriers for providing smoking cessation support and to explore these attitudes in‐depth through qualitative synthesis.

**Methods:**

The online databases AMED, EMBASE, Medline, PsychINFO, HMIC and CINAHL were searched in March 2015 using terms relating to three concepts: ‘attitudes’, ‘mental health professionals’ and ‘smoking cessation’. Quantitative or qualitative studies of any type were included. Proportions of MHPs' attitudes towards smoking and smoking cessation were pooled across studies using random effects meta‐analysis. Qualitative findings were evaluated using thematic synthesis.

**Results:**

Thirty‐eight studies including 16 369 participants were eligible for inclusion. Pooled proportions revealed that 42.2% [95% confidence interval (CI) = 35.7–48.8] of MHPs reported perceived barriers to smoking cessation interventions, 40.5% (95% CI = 30.4–51.0) negative attitudes towards smoking cessation and 45.0% (95% CI = 31.9–58.4) permissive attitudes towards smoking. The most commonly held beliefs were that patients are not interested in quitting (51.4%, 95% CI = 33.4–69.2) and that quitting smoking is too much for patients to take on (38%, 95% CI = 16.4–62.6). Qualitative findings were consistent with quantitative results, revealing a culture of smoking as ‘the norm’ and a perception of cigarettes as a useful tool for patients and staff.

**Conclusions:**

A significant proportion of mental health professionals hold attitudes and misconceptions that may undermine the delivery of smoking cessation interventions; many report a lack of time, training and confidence as main barriers to addressing smoking in their patients.

## Introduction

The prevalence of smoking among people with mental illnesses and substance use disorders is much higher than in the general population. Smoking rates stand at above 70% for those with severe mental illnesses such as schizophrenia and bipolar disorder 1, 2, 3, are similarly high among individuals with substance use disorders 4, 5 and are also above average for those with common mental disorders, including depression and anxiety 1. People with mental illness or substance use disorders are more likely to be heavier and more dependent smokers 6, 7, and their life expectancy is reduced by up to 20 years 8, 9, mainly because of smoking 7. It is therefore vital that smokers from this population receive effective smoking cessation interventions.

Mental health professionals (MHPs), broadly defined as those who received specialist training to offer services designed to improve an individual's mental health (such as clinical psychologists, psychiatrists, clinical social workers and psychiatric nurses), have a crucial role in reducing tobacco smoking among people with mental illness, as they are best placed to encourage and support smokers to quit 10. Indeed, UK clinical guidelines on smoking cessation in secondary care advise that all health and social care practitioners in in‐patient and community‐based mental health services identify smokers and offer advice and assistance to quit 11. However, smokers with mental illness are less likely to be offered advice and support to quit than those without 12, 13.

One possible barrier to the delivery of smoking cessation intervention is MHPs' attitudes towards and beliefs about smoking and/or smoking cessation among individuals with mental illnesses and substance abuse disorders. For instance, previous research has identified that some hold concerns that patients' mental health or abstinence will suffer 14, 15, 16, 17 and that smokers are unable 18 or unmotivated to quit 19. This is in contrast to evidence that smoking cessation, if not actually benefitting mental health, does not impact mental health negatively in people with or without psychiatric disorders 20, even in life‐long, long‐term smokers 21, and that smokers with mental illnesses are just as likely to want to quit as smokers without 22. MHPs have also been reported to hold permissive attitudes towards smoking, such as the belief that smoking with patients can help build a therapeutic relationship 15, 23, 24 and that allowing patients to smoke ensures a smoother running of wards in in‐patient settings 19.

To implement smoking cessation interventions effectively for people with mental illness and substance abuse disorders, it is necessary to understand the relevant attitudes and beliefs of MHPs who engage with these individuals. Such an understanding would lead to greater insight into the potential barriers to delivering smoking cessation support which, in turn, can then inform strategies to reduce tobacco use. However, to our knowledge, there has been no recent systematic investigation of MHPs' attitudes to smoking cessation to identify possible barriers, if any.

The current systematic review and meta‐analysis aims to synthesize the qualitative and quantitative literature on MHPs' attitudes towards smoking and smoking cessation among people with mental illnesses and/or substance abuse disorders. Specifically, it sought to (a) identify and estimate quantitatively MHPs' attitudes towards smoking and main barriers for providing smoking cessation support such as negative attitudes towards smoking cessation and permissive attitudes towards smoking, and (b) to explore these attitudes towards smoking, smoking cessation and support in‐depth through qualitative synthesis.

## Methods

The current review followed Preferred Reporting Items for Systematic Reviews and Meta‐Analyses (PRISMA) guidelines 25.

### Search strategy

The literature was searched for studies reporting on MHPs' beliefs and attitudes relating to smoking among people with mental illnesses and substance use disorders. An initial search strategy was developed in the databases Medline and Embase, and through the identification of key terms used commonly in the literature.

The main keywords used in the search strategy were structured around three key concepts: ‘attitudes’, ‘mental health professionals’ and ‘smoking’, and customized to each database. The final search was conducted on 17 March 2015 in the following databases: AMED (OVID platform), CINAHL (EBSCO platform), classic + Embase, Embase, HMIC Health Management Information Consortium, Medline and PsycINFO (OVID platform) (see Supporting information, Table S1 for full search strategy and number of records identified). To find further eligible papers, forward and backward citation searches of included studies were also conducted.

### Eligibility screening

Studies were eligible for inclusion if they met the following criteria:

1. The sample was health‐care professionals working in mental health, drug or alcohol treatment (referred to as MHPs throughout).
2. The study measured attitudes/beliefs related to patients' smoking or the delivery of smoking cessation support/advice to patients.
3. It was a primary quantitative or qualitative study published in a peer‐reviewed journal (only baseline data from longitudinal or experimental studies were eligible for inclusion to provide unbiased, background estimates).

Studies were excluded if they were not in the English language, focused only on attitudes towards smoke‐free policies, were based on child and adolescent services (the focus of this review was adult services), if the sample was composed only of health professionals in training or if they were published prior to 2003. This date limit was chosen as this was the year in which the World Health Organization Framework Convention of Tobacco Control (WHO FCTC) was published. The WHO FCTC states that parties to the convention should adopt or implement legislation that promotes training on tobacco control for health workers, community workers and social workers 26. As the FCTC was widely ratified and embraced, with 168 signatories, we judged that the date of its publication would serve as an appropriate marker for a shift in MHPs' attitudes towards tobacco and its treatment and capture the most accurate indication of current attitudes.

An inclusion/exclusion criteria checklist was used to screen located articles for inclusion. To establish the reliability of the checklist, two independent reviewers (K.S. and I.T.) screened a random subset (34%) of papers, yielding excellent reliability (Cohen's kappa = 0.95). Disagreements were resolved through discussion.

### Quality appraisal

Studies were evaluated with the Mixed Methods Appraisal Tool (MMAT), which has good validity and reliability 27, 28, 29. For quantitative studies, the MMAT includes three subsections distinguishing between randomized controlled trials, non‐randomized comparative studies and descriptive studies. For the current review, the ‘descriptive’ subsection was used for the appraisal of all included quantitative studies (including where baseline data had been extracted from pre‐test–post‐test or longitudinal studies), as only baseline data and non‐comparative findings were extracted for the purpose of this review.

### Data extraction and synthesis

Data were extracted by one researcher (K.S.) using a standardized data extraction form to provide consistency, reduce bias and increase validity and reliability 30.

#### Quantitative studies

For quantitative studies, all measures of MHPs' attitudes or beliefs relating to smoking among patients or the delivery of smoking cessation support to patients were extracted. Measures of attitudes towards smoke‐free policies and attitudes reported by patients were not extracted. Extracted measures deemed to be reflective of the same beliefs or attitudes were grouped into categories by K.S. and L.S. These categories were organized further into higher‐level grouping on the basis of the types of attitudes or beliefs they represented. The proportion of participants reporting either negative attitudes towards providing smoking cessation support or permissive attitudes towards patients' smoking was extracted for measures within each category. Proportions were extracted systematically using predefined criteria (see Box 1). If relevant data were not included in published reports, authors were contacted to request data.

**Table:** 

**Box 1.** Guidelines for the extraction of proportions	
Response option	Method
Dichotomous (e.g. yes/no)	The proportion of participants choosing either ‘yes’ or ‘no’, depending on which indicated a negative attitude
Yes/unsure/no	The proportion of participants choosing either ‘yes’ or ‘no’, depending on which indicated a negative attitude
4‐point scale	The proportion of participants choosing ‘3’ or ‘4’ OR the proportion of participants choosing ‘1’ or ‘2’, depending on which indicated a negative attitude
5‐point scale	The proportion of participants choosing ‘4’ or ‘5’ OR the proportion of participants choosing ‘1’ or ‘2’, depending on which indicated a negative attitude
6‐point scale	The proportion of participants choosing within the range of 4–6 OR the proportion of participants choosing within the range of 1–3, depending on which indicated a negative attitude
7‐point scale	The proportion of participants choosing within the range of 5–7 OR the proportion of participants choosing 1–3, depending on which indicated a negative attitude
10‐point scale	The proportion of participants responding within the range of 1–4 OR the proportion of participants responding within the range of 7–10, depending on which indicated a negative attitude
100‐point scale	The proportion of participants responding within the range of 1–40 OR the proportion of participants responding within the range of 70–100, depending on which indicated a negative attitude

Where more than one measure from a single study was included in a category, the mean proportion across measures was calculated prior to pooling. Pooling of proportions within each category and within the higher‐level groups was carried out in Stata version 13 using standard methodology 31. Proportions were first transformed using the Freeman–Tukey double arcsine method, pooled with a random effects meta‐analysis with the ‘metan’ command to account for heterogeneity, and then back‐transformed to proportions. Heterogeneity was assessed with the *I* ^2^ statistic 32. Only categories for which data were available from two or more studies were included in the data synthesis to allow for confidence in the accuracy of the findings.

Additionally, a meta‐regression was conducted to examine whether study characteristics were predictive of study outcomes using the ‘metareg’ command in Stata. Following convention 33, only outcomes with data from at least 10 studies were analysed. Study‐level covariates included in the analysis, thought to potentially influence outcomes, were year of publication (pre‐2010/2010‐onwards, based on a median split), country (US/non‐US) and service type (mental health services/drug and alcohol services). All study‐level covariates were entered into the same model concurrently, and values were adjusted for multiple comparisons.

#### Qualitative studies

All text contained in the ‘Results’ or ‘Findings’ section was extracted from published reports and analysed using thematic synthesis. First, textual data were coded line‐by‐line and were then categorized by similarity to generate descriptive themes occurring across included studies 34. Line‐by‐line coding and theme generation was carried out independently by two coders (K.S. and I.T.) and agreed in discussion to establish reliability. As some qualitative studies included interview questions focused on smoke‐free policies, and/or included data gathered from interviews with patients, lines in which findings were related explicitly to smoke‐free policy or attributed to patients were not included in the coding.

## Results

### Search results

**Figure 1:**
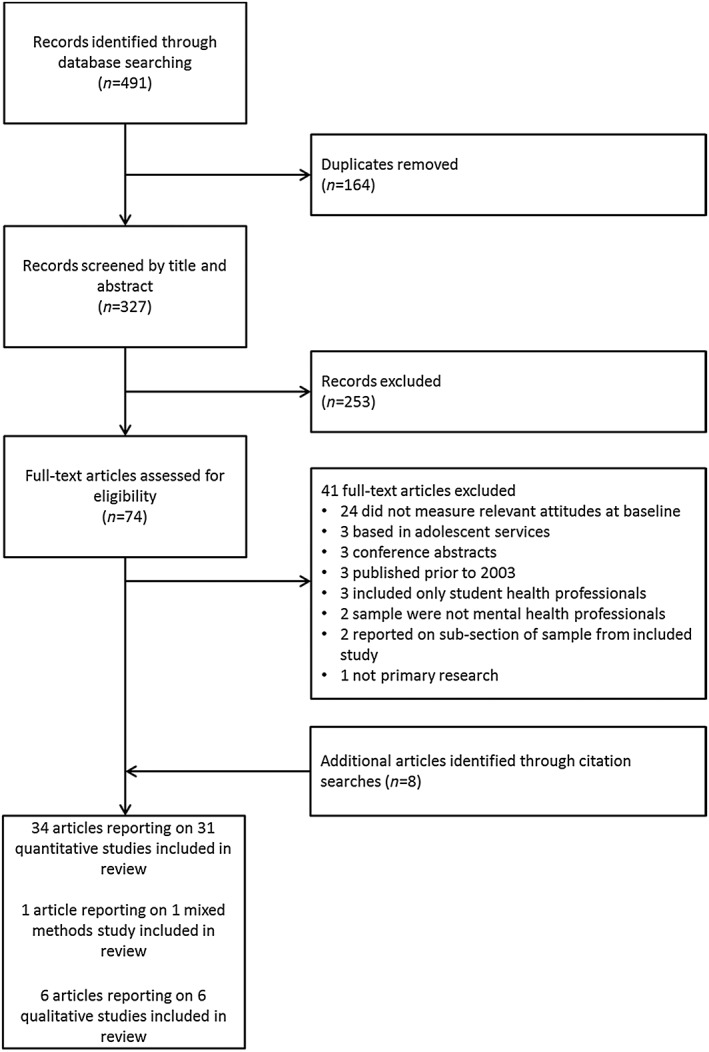
Preferred Reporting Items for Systematic Reviews and Meta‐Analyses (PRISMA) flow diagram

Database searches yielded a total of 327 records after the removal of duplicates. After full‐text screening, 33 papers met inclusion criteria. Forward/backward citation searches yielded an additional eight papers, resulting in a total of 41 papers reporting on 38 studies being included in this review (Fig. 1).

Of the included studies, 31 used a quantitative methodology, six were qualitative and one employed a mixed‐methods approach. The total number of participants across quantitative studies (including the mixed‐methods study) was 16 215. One qualitative study 35 did not report a sample size; across the remainder the total sample size was 154, resulting in a total sample of 16,369 participants across all included studies. The majority of studies recruited MHPs from a range of professional roles (including nurses, psychiatrists and clinical psychologists) were set in a mental health‐care context and conducted in the United States (see Table 1 for details).

**Table 1:** Characteristics of included studies.

*First author (Reference* **)**	*Country*	*Study design*	*Data collection method*	*Sample*	*Recruitment/setting*	*Sample size*	*Response rate*
Akpanudo 49	USA	Cross‐sectional survey	Mailed self‐report questionnaire	Clinical psychologists	Random sample selected from the National Register of Health Service Providers in Psychology	352	57%
Amole 50	USA	Pre‐test–post‐test	Online self‐report questionnaire	Psychiatric nurses	Convenience sample in Georgia, USA, obtained through the American Nurses Crendentialling Centre	201	Not reported
Ashton 39	Australia	Mixed methods	Mailed self‐report questionnaire with closed and open response options	Range of staff	Government and non‐government mental health services in Adelaide, South Australia	324	60%
Brown 51	USA	Pre‐test–post‐test	Mailed self‐report questionnaire	Programme/clinical directors of substance use disorder treatment programmes	Stratified random sample of substance use disorder treatment programmes licensed by the New York State Office of Alcoholism and Substance Abuse Services	285	81.9%
Connolly 24	New Zealand	Cross‐sectional survey	Online self‐report questionnaire	Psychiatric nurses	Invitations for nurses working in in‐patient or community mental health services distributed via the newsletter for a government organization involved with work‐force development across the mental health sector	104	17%
Cookson 53	UK	Cross‐sectional survey	Self‐report questionnaire (administered on site)	Range of staff	Convenience sample from four community drug treatment services associated with the South London and Maudsley NHS Foundation Trust	145	97%
Dickens 23; Stubbs 52	UK	Cross‐sectional survey	Mailed questionnaires	Range of staff	A single large, charitable status, psychiatric hospital in Northampton, UK	599	40.7%
Dwyer 54	Australia	Cross‐sectional survey	Mailed self‐report questionnaire	Psychiatric nurses	Random sample of endorsed mental health nurses selected from the Queensland Nursing Council register	289	28.9%
Fuller 55	USA	Cross‐sectional survey	Self‐report questionnaire (method of administration not reported)	Range of staff	Drug abuse treatment programmes participating in the National Drug Abuse Treatment Clinical Trials Network	3786	71%
Gifford 41	USA	Qualitative	Semi‐structured interviews	Range of staff	15 residential substance abuse treatment programmes	25	N/A
Glover 36	New Zealand	Qualitative	Semi‐structured interviews (face‐to‐face or telephone)	Range of staff	Invited ‘key informants’ including managers and workers in mental health and drug and alcohol services and smoke‐free coordinators and cessation providers within district health boards	61	42%
Guo 56	Taiwan	Cross‐sectional survey	Self‐report questionnaire (method of administration not reported)	Nurses	Two community psychiatric hospitals providing in‐patient and out‐patient care	199	79.6%
Guydish 57	USA	Pre‐test–post‐test	Self‐report questionnaire (administered on site)	Range of staff	All state‐certified addiction treatment programmes in New York State excluding prevention, education, short‐term, hospital‐based, criminal justice and adolescent programmes invited to participate	235	92%
Himelhoch 58	USA	Cross‐sectional survey	Self‐report questionnaire (administered on site)	Primarily psychiatrists and master's level therapists	Nine government‐funded community mental health services in Maryland, USA (only clinicians present on day of recruitment invited)	95	100%
Hunt 38	USA	Cross‐sectional survey	Questionnaire administered via phone, fax, mail or e‐mail	Clinic directors, medical directors, counselling supervisors, head nurses and clinic owners	Representative sample of out‐patients substance abuse treatment facilities selected from the Substance Abuse and Mental Health Services Inventory of Substance Abuse Treatment Services (one person in a leadership position from each clinic)	405	Not reported
Johnson 59	Canada	Cross‐sectional survey	Self‐report questionnaire (administered on site)	Range of staff	8 mental health teams within Vancouver Community Mental Health Services and 14 contracted community agencies	282	32–38%
Keizer 60	Switzerland	Cross‐sectional survey	Self‐report questionnaire (method of administration not reported)	Range of staff	Single public psychiatric hospital in Geneva, Switzerland	155	72.4%
Knudsen 61; Knudsen 62	USA	Longitudinal survey	Self‐report questionnaire administered via phone	Service administrators	Publicly and privately funded substance abuse treatment programmes, and therapeutic communities, identified through prior participation in the National Treatment Centre Study	897	85.2%
Knudsen 63; Knudsen 64	USA	Cross‐sectional survey	Mailed self‐report questionnaire	Counsellors	Publicly and privately funded substance abuse treatment programmes, and therapeutic communities, identified through prior participation in the National Treatment Centre Study	2127	55.5%
Lawn 35	Australia	Qualitative	Participant observation (ethnography) and open‐ended interviews	Range of staff	Two psychiatric hospitals in Queensland and South Australia	Not reported	NA
Leffingwell 65	USA	Cross‐sectional survey	Mailed self‐report questionnaire	Clinical psychologists	Clinical psychologists identified through a public listing of all licensed psychologists in Oklahoma, USA	167	34.7%
McCool 66	USA	Cross‐sectional survey	Self‐report questionnaire administered via phone, fax or mail	Programme leaders (clinic directors, medical directors, supervising counsellors and head nurses)	All out‐patient methadone clinics in the USA (identified from lists of US methadone providers from the Food and Drug Administration and the Center for Substance Abuse Treatment). One programme leader per clinic invited to participate.	408	58.4%
McNally 14	UK	Cross‐sectional survey	Mailed self‐report questionnaire	Range of staff	Three NHS Trusts in London, Staffordshire and Ipswich, UK	837	46%
Miller‐Thomas 67	USA	Cross‐sectional survey	Mailed self‐report questionnaire	Range of staff	12 substance abuse treatment programmes: range of perinatal, Veteran's Affairs, hospital‐based and community‐based	376	85.3%
Morris 40	USA	Qualitative	Focus groups	Range of staff	Representative sample of urban and rural regions in the public mental health system in Colorado, USA	19	NA
Praveen 68	UK	Cross‐sectional survey	Self‐report questionnaire (administered on site)	Range of staff	In‐patient mental health units in Birmingham, Buckinghamshire and central London, UK	308	68.4%
Price 19	USA	Cross‐sectional survey	Mailed self‐report questionnaire	Psychiatrists	Invitations to all community mental health centres with Ohio Department of Mental Health certification	80	53%
Ratschen 16	UK	Cross‐sectional survey	Mailed self‐report questionnaire	Range of staff	All in‐patient mental health units within a single NHS Trust	459	68%
Ratschen 43	UK	Qualitative	Semi‐structured interviews	Range of mental health professionals	Two acute adult mental health wards in a single mental health trust	16	NA
Richter 42	USA	Qualitative	Semi‐structured interviews	Range of staff	Eight drug treatment facilities in a metropolitan area of the Midwestern United States	33	NA
Robson 69	UK	Cross‐sectional survey	Mailed self‐report questionnaire	Psychiatric nurses	Convenience sample from workforce of a single, large NHS Mental Health Trust	585	52%
Sharp 70	USA	Cross‐sectional survey	Electronic self‐report questionnaire	Psychiatric nurses	Sample of nurses selected from members of the American Psychiatric Nurses' Association	1365	31.6%
Sidani 71	USA	Cross‐sectional survey	Mailed self‐report questionnaire	Counsellors	Nationally representative random sample of clinical mental health counsellors identified through membership with the American Mental Health Counsellors Association	330	53.1%
Steiner 72	USA	Cross‐sectional survey	Mailed self‐report questionnaire	Range of staff	Single mental health centre in Connecticut, USA, providing in‐patient and out‐patient services	175	87%
Walsh 17	Australia	Cross‐sectional survey	Mailed self‐report questionnaire	Range of staff	Alcohol and drug treatment agencies identified from the Australian Directory of Alcohol and Other Drug Services and from directories of treatment agencies in all states and territories of Australia. One unit manager and one other staff member per unit invited to participate	417	51.6%
Weinberger 73	USA	Cross‐sectional survey	Self‐report questionnaire (method of administration not reported)	Range of staff	Single mental health centre in Connecticut, USA	34	53%
Williams 37	USA	Pre‐test–post‐test	Self‐report questionnaire (administered on site)	Range of staff	Attendees at a training course focused on training mental health treatment providers to address tobacco dependence	71	NA
Wye 74	Australia	Cross‐sectional survey	Mailed self‐report questionnaire	Nurse managers	All publicly funded psychiatric in‐patient units in New South Wales, Australia. One nurse manager per unit invited to participate	123	94%

### Quality appraisal

All studies included stated clear research questions or objective, and in all but one study (which did not provide the requisite information in the published report 36), data were judged to be sufficient to address the stated research questions or objectives (however, this study provided relevant for the current review and was therefore included here).

#### Quantitative studies (including the quantitative aspect of the mixed‐methods study)

Twenty‐two quantitative studies were judged to have used an adequate random (stratified or non‐stratified) sampling strategy. The remainder used convenience or unclear sampling. For most studies (*n* = 30) the sample representativeness could not be determined, either because the inclusion/exclusion criteria were unclear or because reasons for non‐response or differences between responders and non‐responders were unknown. Two studies were judged to have unrepresentative samples: in one study the sample was attendees at a smoking cessation training programme 3, and in the other the authors stated that their sample was not representative 38. The majority of studies (*n* = 28) used established measures which were clearly reflective of the variable of interest. For the remainder, the source and/or wording of measures was unclear. Fewer than half (*n* = 15) of the studies had a response rate of ≥ 60%.

#### Qualitative studies (including the qualitative aspect of the mixed‐methods study)

All qualitative studies used data sources (i.e. participants/recruitment settings) that were relevant to the research question, and used relevant approaches to data collection and analysis. Four of the seven qualitative studies discussed their findings in consideration of the research context, but only one explicitly considered the researchers' influence on their findings.

### Synthesis of quantitative findings

Fourteen categories of attitudes were measured in at least five of the studies included and were therefore considered to be reliable and replicable. These categories were organized into three higher‐level groups: ‘perceived barriers to providing support’ (three categories), ‘negative attitudes towards smoking cessation’ (seven categories) and ‘permissive attitudes towards smoking’ (four categories).

The most frequently measured categories were ‘lack of knowledge/training/skills in providing smoking cessation treatment’, ‘quitting smoking might have a negative impact on symptoms/recovery’, ‘smoking cessation is not a priority’, ‘patients are not interested in quitting’ and ‘lack of time to provide smoking cessation treatment’ (see Table 2 for all categories and example measures). For some categories, suitable data could not be extracted from at least two studies, and therefore these categories were not included in the quantitative analysis.

**Table 2:** Categories of attitudes/beliefs measured in five or more included studies.

Category	Example measures	No. of studies measuring category
**Perceived barriers to the provision of smoking cessation support**		
Lack of knowledge/training/skills is a barrier to providing treatment	‘Mental health nurses do not have the appropriate skills to help a smoker with mental illness stop smoking’	16^b^
Lack of time is a barrier to providing smoking cessation treatment	‘I don't have enough time as a healthcare provider to deal with tobacco use’	12^b^
Low confidence in ability to address patients' smoking	‘How confident are you in your ability to counsel smokers who are interested in quitting smoking?’	12^b^

**Negative attitudes towards smoking cessation**		
Quitting smoking might have a negative impact on symptoms/recovery	‘Quitting smoking would make other mental health symptoms worse’	15
Smoking cessation treatment is not a priority	‘Smoking and tobacco use are not important issues in the successful treatment of other substance abuse problems’	14
Patients are not interested in quitting	‘Most patients who smoke aren't interested in giving up smoking’	13^b^
Smoking cessation treatment is not part of own role	‘Do you believe it is part of your role to help clients to become smoke‐free?’	11^b^
Providing smoking cessation treatment is not part of the role of drug treatment/mental health services	‘How involved should mental health services be in helping people with a mental illness to quit smoking or reduce their consumption?’	10^b^
Patients who smoke should not be encouraged/given assistance to quit	‘Do you think that patients who smoke should be encouraged/helped to stop or cut back?’	6^a^, ^b^
Smoking cessation intervention is not effective	‘Pessimism about the effectiveness of stop smoking interventions is a barrier’	5^b^

**Permissive attitudes towards smoking** b		
Smoking is an important coping mechanism	‘Smoking helps clients cope with the stress in their lives’	6^a^, ^b^
Smoking with patients helps to establish a therapeutic relationship	‘Sometimes it is useful for staff to smoke with a patient to build rapport/trust’	5^b^
Quitting smoking is too much for patients to take on alongside their other issues	‘Clients should not be encouraged to give up smoking, as they have enough to cope with’	5^b^
Smoking is a personal choice	‘Clients can decide for themselves about quitting’	5^a^, ^b^

a Not included in meta‐analysis as data could not be extracted from ≥ 2 studies.

b Not included in meta‐regression as data could not be extracted from ≥10 studies.

Because there was significant heterogeneity across measures within all categories (all *I* ^2^ ≥ 94.7, all *P*s < 0.001), data were pooled using a random‐effects meta‐analysis (see Table 3).

**Table 3:** Pooled proportions within included categories of beliefs/attitudes.

Category	Studies included in analysis	Pooled proportion (95% CI)	Range	Pooled frequencies	I^2^ (%)
**Perceived barriers**	17, 19, 49, 51, 53, 58, 61, 62, 63, 64, 70, 71	**42.2 (35.7–48.8)**	**16.2–61.9%**	**2588/5870**	**95.5**
Lack of knowledge/training/skills	17, 19, 49, 63, 64, 70, 71, 73	35.8 (24.3–48.2)	17.1–61.9%	1902/5382	98.6
Lack of time	17, 19, 51, 58, 61, 62, 63, 64, 71	35.1 (24.4–46.7)	5.1–56.0%	1442/4129	97.8
Low confidence	17, 19, 49, 53, 70, 71	31.0 (20.1–43.1)	16.2–49.7%	1016/2530	97.1

**Negative attitudes**	17, 19, 24, 37, 38, 49, 39, 51, 53, 55, 58, 60, 61, 62, 63, 64, 65, 66, 70, 71, 74	**40.5 (30.4–51.0)**	**13.5–76.6%**	**4027/11 184**	**99.1**
Patients are not interested	17, 19, 49, 58, 70, 71, 74	51.4 (33.4–69.2)	19.9–76.6%	1490/2556	98.6
Intervention is not effective	17, 19, 37, 49, 71	27.6 (13.8–44.2)	2.5–55.1%	444/1217	97.1
Not a priority	19, 38, 49, 39, 53, 60, 61, 62, 63, 64, 70, 74	26.5 (19.5–34.2)	2.5–59.9%	1938/5876	97.2
Negative impact on symptoms/recovery	17, 19, 37, 38, 49, 51, 58, 61, 62, 63, 64, 71	24.4 (14.8–35.4)	5.0–70.5%	1110/4799	98.4
Not part of own role	24, 37, 51, 65, 71	18.0 (1.8–45.7)	2.1–72.5%	182/936	98.8
Not part of the role of services	38, 39, 55, 60, 66, 74	17.4 (11.6–24.0)	8.4–44.7%	868/4988	94.7

**Permissive attitudes**	17, 23, 52, 24, 38, 51, 69, 74	**45.0 (31.9–58.4)**	**22.8–57.9**	**1055/2471**	**97.7**
Quitting smoking is too much	17, 38, 51, 69, 74	38.0 (16.4–62.6)	8.3–57.9	607/1802	99.1
Helps to establish therapeutic relationship	17, 23, 52, 24, 69, 74	33.5 (17.6–51.6)	14.6–54.3%	594/1797	98.3

#### Perceived barriers

Around four of 10 participants reported perceived barriers to offering smoking cessation intervention, with lack of knowledge or training being the most prevalent perceived barrier, followed by lack of time and low confidence.

#### Negative attitudes to cessation

A similar proportion reported negative attitudes related to smoking cessation. The most commonly held beliefs were that patients are not interested in quitting smoking and that smoking cessation interventions are not effective. The least prevalent beliefs were that delivering smoking cessation intervention is not part of MHPs' role or that of mental health/drug and alcohol services.

#### Permissive attitudes to smoking

Nearly half of MHPs held permissive attitudes towards patients' smoking, with more than a third reporting the belief that quitting smoking is too much for patients to take on and that smoking with patients helps to establish a therapeutic relationship or build rapport.

#### Meta‐regression

Meta‐regression was conducted on only a subsection of outcomes (see Table 2) and revealed no significant associations of study characteristics with proportions for higher‐level groupings. However, in the analysis of individual categories there was a significant association between study country and the proportion of respondents who endorsed the view that quitting smoking might have a negative impact on symptoms and recovery. Controlling for other study‐level covariates, MHPs in studies from the United States were less likely than those from other countries to be worried about the negative impact of smoking cessation on mental health, with an absolute difference in proportions of 30.3% [95% confidence interval (CI) = 3.1–69.6, *P* < 0.05].

### Synthesis of qualitative findings

Seven studies were included in the qualitative synthesis. The thematic synthesis yielded five main/recurring themes: (1) beliefs about patients quitting smoking; (2) barriers to the provision of smoking cessation treatment; (3) attitudes to the provision of smoking cessation treatment; (4) acceptance of patients' smoking; and (5) smoking as a useful tool. Table 4 shows the subthemes identified within each of these alongside illustrative quotes.

**Table 4:** Themes and subthemes identified in qualitative synthesis.

*Theme*	*Subtheme*	*Study references*	*Illustrative quotes*
Beliefs about patients quitting smoking	Negative perceptions of patients' motivation/ability to quit	35, 37, 32, 31, 36, 39, 38	‘Some directors and staff members 9 and only 1 client cited that clients do not want to quit… ‘But the reality is most of our adult smokers could care less. I mean they're not interested in quitting smoking’ 38
			‘Providers and consumers both voiced negative expectations regarding the ability of persons with mental illnesses to quit smoking, but providers made these comments more frequently’ 36
	Concerns about the negative consequences of quitting	37, 32	‘Some wondered whether attempting to provide SC treatment in SRTPs could jeopardize patients' sobriety’ 37
			‘Helping service users to quit smoking was ‘too hard’…, and there was a fear of patients becoming violent’ 32
Barriers to the provision of smoking cessation treatment	Lack of opportunity to provide treatment	35, 37, 32, 36, 39, 38	‘Providers cited a lack of clinical resources such as smoking cessation groups and financial resources [both patient and system] to pay for the treatment’ 36
			‘Time restraints mean other issues increase in priorities’ 35
	Lack of capability to provide treatment	32, 36, 39, 37	‘The participants' skills and knowledge relating to smoking and nicotine dependence treatment seemed lacking’ 39
			‘One respondent suggested that there was a ‘total lack of knowledge’ about the importance of, or the need for, service users to stop smoking’ 32
	Health professionals' behaviour	37, 32, 31, 36, 38	‘In the locked settings, clients and staff spent much time in direct contact, often in the smoking area with the majority of clients and staff smoking, with staff acting as social role models for clients at such times’ 31
			‘Far from encouraging cessation, some staff enabled smoking by “offering cigarettes” with ‘nurses who purchase cigarettes [for the service users], even out of their own money at times’ 32
Attitudes to the provision of smoking cessation treatment	It is important	35, 37, 32, 36, 39, 38	‘Providers identified tobacco cessation for persons with mental illnesses as a promising or emerging evidence‐based practice and strongly supported integrating tobacco cessation services in mental health settings as a clinical priority’ 36
	It is not a priority	35, 37, 32, 36, 39, 38	‘Another difference reported [between smoking and other drug use] was that smoking was not a focus or a high priority in drug treatment’ 38
			‘An ‘unwillingness to place nicotine addiction high enough to warrant the same attention as other addictions’ led to a ‘low need to quit’ 32
	It is not part of own role	35, 37, 32, 38	‘Several participants said that SC counseling was not in their job description and was outside their scope of work’ 37
			‘The capacity to provide cessation support was perceived to be limited by exclusion of the requirement to possess the skill or deliver support in employee job descriptions’ 32
	Negative beliefs about providing treatment	37, 32, 36, 38	‘Participants stated concerns that “trying to force patients to quit” might make them leave the programme’ 37
			‘As one provider put it, “the problem is that there isn't actually evidence that it [cessation strategies] works”’ 36
	It is dependent upon the patient	35, 37, 39, 38	‘Most participants expressed discomfort with advising uninterested patients to quit, indicating interventions were typically presented to only patients who explicitly asked for help. One participant described the process as, “If they say they don't want to stop smoking, they get told about the dangers and that's it”’ 37
Acceptance of patients' smoking	Culture of smoking	37, 32, 31	‘A number of staff beliefs and practices supported a “culture of smoking”, with “smoking as the norm”’ 32
	It is a patients' right/personal choice	35, 37, 32	‘I believe people should have a choice if they smoke or not’ 39
	Smoking is a ‘core need’	31, 36, 39	‘Several providers commented that “they [mental health consumers] don't care how much they spend on cigarettes. Their cigarettes are so important to them, it doesn't matter”’ 36
			‘Clients and staff focused much attention on ensuring the supply of cigarettes as a core need for clients’ 31
Smoking as a useful tool	For patients	32, 31, 36, 39, 38	‘Respondents generally viewed smoking as an important coping mechanism for patients—providing a way to deal with stress’ 39
			‘In the locked ward I don't think there's much in the way of one‐to‐one therapeutic activity that happens. It's a kind of, “Let's wait for the medication to work”. There's just nothing to do. The only normal thing to do at the time is to smoke’ 31
	For staff	37, 32, 31, 36	‘Some respondents believed that smoking enabled positive social experiences, which helped staff develop rapport with service users’ 32
			‘Moreover, in some settings, such as psychiatric hospitals, consumers earned smoking privileges as a behavioural reward’ 36

#### Beliefs about patients quitting smoking

The most apparent subtheme within this theme was negative perceptions of patients' ability and motivation to quit, which was identified in all studies. This perception appeared to arise from beliefs that patients would not be interested in quitting smoking 39, 40, 41, 42, that patients would be unable to quit smoking successfully 35, 36, 40 and the view that smoking cessation would be too much for patients to cope with alongside their other issues 36, 39, 41, 42, 43. A minority of studies reported concerns about the negative consequences of quitting smoking, such as concerns about a negative impact on psychiatric symptoms/abstinence 36, 41, the potential effects on medication 36 and a fear that smoking cessation may make patients violent 36.

#### Barriers to the provision of smoking cessation treatment

A perceived lack of opportunity to provide smoking cessation support was evident 36, 39, 40, 41, 42, 43. This generally arose from a perceived lack of treatment resources either on‐site or to refer patients to off‐site 36, 40, 41, 42 and a lack of guidance for implementing smoking cessation treatment alongside treatment for patients' primary disorders 36, 41, 42, 43. In addition, a lack of staff 36, 41, staff time 36, 39, funding 36, 40 and support from management 36, 41 were identified as further barriers to providing smoking cessation treatment.

In a number of studies, staff did not appear to feel that they had the capability to treat nicotine dependence effectively 36, 40, 41, 43. This arose both from a lack of knowledge about nicotine dependence and pharmacological treatments 36, 40, 41, 43 and from a perceived need for specialized training in the provision of smoking cessation support to people with mental illnesses and/or drug and alcohol disorders 40, 41, 43.

The behaviour of MHPs was identified commonly as a barrier to addressing smoking among patients 35, 40, 41, 42. This tended to relate to professionals' smoking status, as staff smoking was viewed as providing a bad example to patients 35, 36, 40, 41, 42 and could potentially undermine motivation to address patients' smoking (e.g. because it would be hypocritical) 41. In addition, professionals' role in facilitating patients' smoking (i.e. by providing lights and/or cigarettes) was a further potential barrier to providing smoking cessation treatment 35, 36.

#### Attitudes to the provision of smoking cessation treatment

Smoking cessation generally was viewed to be important for patients 36, 39, 40, 41, 42, 43, primarily because of the potential to improve patients' health 39, 41, 42, 43 and reduce their financial burden 39, 42. Two studies reported that staff felt smoking cessation treatment should be integrated into patients' care 39, 43. Despite this positive attitude to the provision of smoking cessation treatment, results from the majority of studies indicated that the provision of smoking cessation treatment was not viewed as a priority in the context of treatment for patients' primary disorders [36,39–43.] For instance, some staff thought that treating tobacco dependence was less important than treating addictions to alcohol or illicit drugs, as cigarettes are not illegal or as harmful to health 36, 42. In some cases, the provision of smoking cessation treatment was seen to be dependent upon the patient; for instance, if they showed an interest in quitting 39, 41, 42, 43 or displayed any negative health effects of smoking 39.

The provision of smoking cessation treatment was often not seen as part of MHPs' role 36, 39, 41, 42, and in many cases staff held negative beliefs about smoking cessation treatment 36, 40, 41, 42. For instance, some reported the belief that smoking cessation treatment is not effective 40, 41, while others had concerns that encouraging smoking cessation might drive patients away 36, 41.

#### Acceptance of smoking

Overall, a general acceptance of patients' smoking by MHPs could be noted. This seemed to stem from the view that there was a long‐standing ‘culture of smoking’ in in‐patient mental health and drug and alcohol services 35, 36, 41, alongside the belief that it is a patient's right/personal choice to smoke 36, 39, 41. Further, some staff felt that smoking was a ‘core need’ for their patients—something essential that they could not be without 35, 40, 43.

#### Smoking as a useful tool

Across studies, MHPs viewed cigarettes and smoking as useful tools in managing patients 35, 36, 40, 41, 42, 43. Patients were seen to use smoking as a form of self‐medication (e.g. to cope with abstinence from other addictive drugs or with psychiatric symptoms) or stress‐relief 35, 36, 40, 42, 43, to relieve boredom 35, 40 and facilitate socializing 40. Smoking was also viewed as a useful tool to be used by staff, either through smoking with patients to build rapport 35, 36, 40, 41, by using cigarettes as a behavioural incentive/reward 35, 36, 40 or using cigarettes to keep patients calm and minimize aggression 35.

## Discussion

We identified 38 relevant studies investigating MHPs' attitudes towards smoking and smoking cessation among people with mental illnesses and substance abuse disorders, most of which were quantitative rather than qualitative. A significant proportion of MHPs held negative attitudes towards smoking cessation and permissive attitudes towards smoking, and perceived a number of barriers to providing smoking cessation treatment. Meta‐regression analyses revealed no associations between attitudes and year of publication or type of service, but showed that MHPs in US‐based studies were less likely to hold concerns about the negative impact of smoking cessation on symptoms and recovery. The thematic synthesis of qualitative studies was consistent with the quantitative results, and in addition revealed a richer insight into MHPs' views on patients' smoking, particularly in the context of in‐patient services. Cigarettes were viewed as a useful tool for both patients and staff, and the smoking behaviour of staff was identified as a barrier to providing effective smoking cessation support. A general acceptance of patients' smoking was also apparent in the qualitative findings, with a culture of smoking being seen as the ‘norm’ in some services.

This synthesis of the existing literature examining MHPs' attitudes towards smoking and smoking cessation among patients reveals that a significant minority hold attitudes and beliefs that may prevent them from delivering smoking cessation support. The relationship between attitudes/beliefs, intentions and behaviour is well established (e.g. 44, 45), and health professionals are no exception 46. This review highlights the potential for such attitudes to present a barrier to integrate smoking cessation interventions effectively into treatment for mental illnesses and substance use disorders. In addition, this review identified a number of target misconceptions that are inconsistent with the scientific literature. For instance, the belief that patients are not interested in quitting is contradicted by evidence that people with mental illnesses are just as likely to want to quit as those without 22. Similarly, concerns about the negative impact of quitting on patients' symptoms or recovery are not supported by research 20, 21.

It is encouraging that the current review identified that most MHPs felt that it was part of their role to address patients' smoking, even though a minority of approximately one‐fifth felt that it was not part of their role. Further, the qualitative findings indicated that most felt that smoking cessation was important, suggesting that MHPs view the provision of smoking cessation treatment positively. However, despite this generally positive attitude towards providing support, MHPs' reported beliefs about patients' ability and/or motivation to quit, and perceptions of their own ability to successfully deliver interventions and intervention effectiveness which may prevent them from providing support. Most notably, more than half of MHPs in this review reported that patients are not interested in quitting smoking and almost 40% felt that quitting smoking was too much for patients to take on, suggesting that one of the main barriers to the delivery of smoking cessation support may be implicit beliefs about patients, and a tendency to shift responsibility for smoking cessation to patients alone. In agreement with previous work 18, these findings highlight a need for greater prioritization of smoking cessation treatment in mental health and substance abuse care, a need for specialist training in smoking cessation interventions and broader education to challenge misconceptions about smoking cessation in the context of mental health and drug and alcohol treatment. One notable policy change recently instigated in the United Kingdom that may prove effective is the introduction of smoke‐free policies 47; other levers which could be pursued to address existing barriers include mandatory smoking cessation training for MHPs and, in the context of private health‐care systems, governmental subsidies for cost‐effective smoking cessation pharmacotherapy. In fact, National Institute for Health and Care Excellence (NICE) guidance on smoke‐free policy indicates that this policy should only be introduced alongside training and systematic identification of smokers and treatment, as introducing such policies by themselves is unlikely to change attitudes 11. In addition, the inclusion of effective smoking cessation medications on the hospitals' formulary will ensure that smokers with and without mental illness have equal access to pharmacotherapy to treat tobacco addiction.

It is unclear why MHPs in the United States were less likely to be concerned about the negative impact of smoking cessation than those from other countries. This may reflect differences in the training provided to MHPs in different countries. However, clinical practice guidelines on smoking cessation from the United States, Australia and the United Kingdom (the two countries that formed the majority of studies not conducted in the United States) do not provide a clear explanation for this divide. While guideline recommendations in the United Kingdom do not cover the topic of smoking cessation, psychiatric symptoms and addiction recovery explicitly, they clearly encourage smoking cessation in this setting 11, and both US 48 and Australian 3 guidelines state that smoking cessation is unlikely to have any adverse effects. The lack of evidence of any recent changes in attitudes to support cessation is also of concern in light of accumulating evidence of the positive impact of cessation on mental health 20, 21, 22, and may indicate a need to disseminate this information to MHPs more effectively.

The main strength of this review is that is provides an up‐to‐date overview of the most prevalent beliefs and attitudes that may present a barrier to the delivery of smoking cessation intervention in mental health and drug and alcohol services. This review also has some limitations. Data for pooled proportions were not available from all studies, and there was significant heterogeneity among studies. However, quantitative results were largely confirmed by the qualitative synthesis, increasing confidence in these findings. The study selection might be biased due to the focus on English language and full‐text publications; however, every effort was made to make the search strategy as inclusive as possible. None the less, relatively little qualitative work was identified, and further studies should explore potential solutions to overcome common barriers and enable the effective integration of stop smoking service provision into mental health care.

In summary, a significant proportion of MHPs appear to hold negative attitudes towards offering smoking cessation advice and support to patients with mental illness and substance use disorders. These present a potential barrier to the successful implementation of smoking cessation interventions in mental health/substance abuse services, and highlight the continued need for dedicated education and training amongst this group of health professionals.

### Declaration of interests

L.S. has received a research grant and honoraria for a talk and travel expenses from a Pfizer, manufacturer of smoking cessation medications.

## Supporting information


**Table S1**. Full search strategy and records identified.

Supporting info item
