# Supplementary material for: A mixed‐method systematic review and meta‐analysis of mental health professionals' attitudes toward smoking and smoking cessation among people with mental illnesses
Source: Addiction. 2016 May 3;111(9):1536–53. doi: 10.1111/add.13387 (PMC5025720; doi:10.1111/add.13387)
Supplement: Supplementary file 1 — Supporting info item [file ADD-111-1536-s001.docx]

**Table S1. Full search strategy and records identified**

| **Ovid AMED** | | | | **Ovid Embase** | | |
| --- | --- | --- | --- | --- | --- | --- |
| ***Search*** | ***Search terms*** | | ***Records (n)*** | ***Search*** | ***Search terms*** | ***Records (n)*** |
| S1 | attitude/ OR attitude*.mp | | 11,483 | S1 | health personnel attitude/ OR attitude/ OR attitude*.mp | 384,264 |
| S2 | opinion*.mp OR “Attitude of health personnel”/ | | 4,389 | S2 | opinion*.mp | 99,732 |
| S3 | belief*.mp | | 2,196 | S3 | belief*.mp | 68,426 |
| S4 | view*.mp | | 5,899 | S4 | view*.mp | 409,991 |
| S5 | perception*.mp | | 6,720 | S5 | perception*.mp | 281,241 |
| S6 | mental health personnel.mp | | 4 | S6 | mental health personnel.mp OR mental health care personnel/ | 1,375 |
| S7 | mental health staff.mp | | 8 | S7 | mental health staff.mp | 317 |
| S8 | psychiatric staff.mp | | 1 | S8 | psychiatric staff.mp | 189 |
| S9 | mental health professional*.mp | | 130 | S9 | mental health professional*.mp | 4,940 |
| S10 | mental health worker*.mp | | 30 | S10 | mental health worker*.mp | 692 |
| S11 | mental health services/ OR mental health service*.mp | | 1,565 | S11 | mental health service/ OR mental health service*.mp | 49,336 |
| S12 | community mental health services/ OR community mental health service*.mp | | 757 | S12 | community mental health service*.mp | 1,025 |
| S13 | drug and alcohol service*.mp | | 3 | S13 | drug and alcohol service*.mp | 140 |
| S14 | substance abuse service*.mp | | 4 | S14 | substance abuse service*.mp | 406 |
| S15 | psychiatric nurse*.mp | | 27 | S15 | psychiatric nurse*.mp | 2,180 |
| S16 | mental health nurse*.mp | | 17 | S16 | mental health nurse*.mp | 1,762 |
| S17 | psychiatrist*.mp | | 222 | S17 | psychiatrist/ OR psychiatrist*.mp | 34,634 |
| S18 | key worker*.mp | | 33 | S18 | key worker*.mp | 286 |
| S19 | clinical psychologist*.mp | | 52 | S19 | clinical psychologist*.mp | 1,875 |
| S20 | smoking/ OR smoking.mp | | 1,038 | S20 | smoking/ OR smoking.mp | 309,762 |
| S21 | smoking cessation/ OR smoking cessation.mp | | 257 | S21 | smoking cessation.mp OR smoking cessation/ | 44,346 |
| S22 | smoking ban.mp | | 0 | S22 | smoking ban.mp OR smoking ban/ | 1,282 |
| S23 | smokefree.mp | | 0 | S23 | smokefree.mp | 299 |
| S24 | smoke-free.mp | | 8 | S24 | smoke free.mp | 2,535 |
| S25 | smoke free.mp | | 8 | S25 | smoke-free.mp | 2,535 |
| S26 | 1 OR 2 OR 3 OR 4 OR 5 | | 23,299 | S26 | 1 OR 2 OR 3 OR 4 OR 5 | 1,100,130 |
| S27 | 6 OR 7 OR 8 OR 9 OR 10 OR 11 OR 12 OR 13 OR 14 OR 15 OR 16 OR 17 OR 18 OR 19 | | 1,989 | S27 | 6 OR 7 OR 8 OR 9 OR 10 OR 11 OR 12 OR 13 OR 14 OR 15 OR 16 OR 17 OR 18 OR 19 | 89,123 |
| S28 | 20 OR 21 OR 22 OR 23 OR 24 OR 25 | | 1,039 | S28 | 20 OR 21 OR 22 OR 23 OR 24 OR 25 | 309,905 |
| S29 | 26 AND 27 AND 28 | | 3 | S29 | 26 AND 27 AND 28 | 148 |
| **Ovid Medline** | | | | **Ovid PsychINFO** | | |
| ***Search*** | | ***Search terms*** | ***Records (n)*** | ***Search*** | ***Search terms*** | ***Records (n)*** |
| S1 | | “attitude of health personnel”/ OR attitude/ OR attitude*.mp | 311,393 | S1 | attitudes/ OR health personnel attitudes/ OR attitude*.mp | 348,108 |
| S2 | | opinion*.mp | 75,184 | S2 | opinion*.mp | 43,889 |
| S3 | | belief*.mp | 49,065 | S3 | belief*.mp | 106,161 |
| S4 | | view*.mp | 313,738 | S4 | view*.mp | 248,322 |
| S5 | | perception*.mp | 292,616 | S5 | perception*.mp | 375,596 |
| S6 | | mental health personnel.mp | 71 | S6 | mental health personnel.mp OR exp mental health personnel/ | 44,387 |
| S7 | | mental health staff.mp | 219 | S7 | mental health staff.mp | 406 |
| S8 | | psychiatric staff.mp | 150 | S8 | psychiatric staff.mp | 303 |
| S9 | | mental health professional*.mp | 3,340 | S9 | mental health professional*.mp | 12,899 |
| S10 | | mental health worker* | 519 | S10 | mental health worker*.mp | 1,558 |
| S11 | | mental health services/ OR mental health service*.mp | 46,149 | S11 | mental health services/ OR Mental health service*.mp | 41,274 |
| S12 | | community mental health services/ OR community mental health service*.mp | 17,014 | S12 | community mental health services/ OR community mental health service*.mp | 7,169 |
| S13 | | drug and alcohol service*.mp | 76 | S13 | drug and alcohol service*.mp | 127 |
| S14 | | substance abuse service*.mp/ OR substance abuse treatment centers/ | 4,910 | S14 | substance abuse service*.mp | 510 |
| S15 | | psychiatric nurse*.mp | 1,904 | S15 | psychiatric nurse/ OR psychiatric nurse*.mp | 3,792 |
| S16 | | mental health nurse*.mp | 1,376 | S16 | mental health nurse*.mp | 1,383 |
| S17 | | psychiatrist*.mp | 17,594 | S17 | psychiatrists/ OR psychiatrist*.mp | 36,085 |
| S18 | | key worker*.mp | 180 | S18 | key worker*.mp | 188 |
| S19 | | clinical psychologist*.mp | 1,104 | S19 | clinical psychologist*.mp | 6,519 |
| S20 | | smoking/ OR smoking.mp | 199,569 | S20 | smoking.mp OR tobacco smoking/ | 40,376 |
| S21 | | smoking cessation.mp OR smoking cessation/ | 26,119 | S21 | smoking cessation.mp OR smoking cessation/ | 11,826 |
| S22 | | smoking ban.mp | 539 | S22 | smoking ban.mp | 257 |
| S23 | | smokefree.mp | 192 | S23 | smokefree.mp | 82 |
| S24 | | smoke free.mp | 2,117 | S24 | smoke free.mp | 893 |
| S25 | | smoke-free.mp | 2,117 | S25 | smoke-free.mp | 893 |
| S26 | | 1 OR 2 OR 3 OR 4 OR 5 | 914,241 | S26 | 1 OR 2 OR 3 OR 4 OR 5 | 904,308 |
| S27 | | 6 OR 7 OR 8 OR 9 OR 10 OR 11 OR 12 OR 13 OR 14 OR 15 OR 16 OR 17 OR 18 OR 19 | 72,004 | S27 | 6 OR 7 OR 8 OR 9 OR 10 OR 11 OR 12 OR 13 OR 14 OR 15 OR 16 OR 17 OR 18 OR 19 | 117,181 |
| S28 | | 20 OR 21 OR 22 OR 23 OR 24 OR 25 | 199,739 | S28 | 20 OR 21 OR 22 OR 23 OR 24 OR 25 | 40,415 |
| S29 | | 26 AND 27 AND 28 | 133 | S29 | 26 AND 27 AND 28 | 108 |
| **Ovid HMIC** | | | | **EBSCO CINAHL** | | |
| ***Search*** | | ***Search terms*** | ***Records (n)*** | ***Search*** | ***Search terms*** | ***Records (n)*** |
| S1 | | attitude*.mp OR attitudes/ | 10,952 | S1 | “attitude*” OR (MH “attitude”) OR (MH “attitude of health personnel”) | 201,044 |
| S2 | | opinion*.mp OR opinions/ | 5,519 | S2 | “opinion*” | 21,934 |
| S3 | | belief*.mp OR beliefs/ | 3,062 | S3 | “belief*” | 29,865 |
| S4 | | view*.mp OR views/ | 23,974 | S4 | “view*” | 62,769 |
| S5 | | perception*.mp | 6,696 | S5 | “perception*” | 84,285 |
| S6 | | mental health personnel.mp | 3 | S6 | (MH “mental health personnel+”) OR “mental health personnel” | 6,959 |
| S7 | | mental health staff.mp | 108 | S7 | “mental health staff” | 187 |
| S8 | | psychiatric staff.mp | 27 | S8 | “psychiatric staff” | 50 |
| S9 | | mental health professional*.mp | 462 | S9 | “mental health professional*” | 1,789 |
| S10 | | mental health worker*.mp | 211 | S10 | “mental health worker*” | 408 |
| S11 | | mental health service*.mp OR mental health services/ | 9,934 | S11 | “mental health service*” OR (MH “mental health services”) | 32,796 |
| S12 | | community mental health service*.mp OR community mental health services/ | 1,031 | S12 | (MH “community mental health services”) OR “community mental health service*” | 8,131 |
| S13 | | drug and alcohol service*.mp | 46 | S13 | “drug and alcohol service*” | 54 |
| S14 | | substance abuse service*.mp OR substance abuse treatment services/ | 776 | S14 | “substance abuse service*” | 160 |
| S15 | | psychiatric nurse*.mp | 690 | S15 | “psychiatric nurse*” | 2,167 |
| S16 | | mental health nurse*.mp OR mental health nurses/ | 931 | S16 | “mental health nurse*” | 2,233 |
| S17 | | psychiatrists/ OR psychiatrist*.mp | 1,562 | S17 | (MH “psychiatrists”) OR “psychiatrist*” | 4,792 |
| S18 | | key worker* | 183 | S18 | “key worker*” | 175 |
| S19 | | clinical psychologist* | 203 | S19 | “clinical psychologist*” | 379 |
| S20 | | smoking/ OR smoking.mp | 8,009 | S20 | (MH “smoking”) OR “smoking” | 61,780 |
| S21 | | smoking cessation.mp OR smoking cessation/ | 2,075 | S21 | (MH “smoking cessation”) OR “smoking cessation” | 16,583 |
| S22 | | smoking ban.mp | 102 | S22 | “smoking ban” | 391 |
| S23 | | smokefree.mp | 93 | S23 | “smokefree” | 127 |
| S24 | | smoke free.mp | 381 | S24 | “smoke free” | 1,150 |
| S25 | | smoke-free.mp | 381 | S25 | “smoke-free” | 1,150 |
| S26 | | 1 OR 2 OR 3 OR 4 OR 5 | 40,801 | S26 | 1 OR 2 OR 3 OR 4 OR 5 | 330,496 |
| S27 | | 6 OR 7 OR 8 OR 9 OR 10 OR 11 OR 12 OR 13 OR 14 OR 15 OR 16 OR 17 OR 18 OR 19 | 13,350 | S27 | 6 OR 7 OR 8 OR 9 OR 10 OR 11 OR 12 OR 13 OR 14 OR 15 OR 16 OR 17 OR 18 OR 19 | 47,345 |
| S28 | | 20 OR 21 OR 22 OR 23 OR 24 OR 25 | 8019 | S28 | 20 OR 21 OR 22 OR 23 OR 24 OR 25 | 61,824 |
| S29 | | 26 AND 27 AND 28 | 21 | S29 | 26 AND 27 AND 28 | 78 |
